# An ATP-associated membrane interface integrating methionine flux with redox-regulated signaling in cancer

**DOI:** 10.3389/fonc.2026.1779365

**Published:** 2026-03-05

**Authors:** Maximo A. Benavides

**Affiliations:** Bio-Medical Science World Corp., Houston, TX, United States

**Keywords:** ATP-dependent membrane processes, cancer metabolism, membrane energetics, methionine metabolism, one-carbon metabolism, post-translational modifications, redox signaling

## Abstract

Methionine dependence and redox-regulated post-translational modifications (PTMs) represent well-characterized and therapeutically relevant features of cancer cell metabolism. Although established amino acid transporters and one-carbon pathways account for methionine uptake and utilization, current models do not fully explain how methionine influx is dynamically integrated with ATP-dependent membrane energetics and redox-sensitive signaling networks in malignant cells. Here, we propose a testable conceptual framework in which a thiol- and methyl-responsive, ATP-associated membrane interface operates at the membrane–metabolism boundary, coupling methionine availability with redox-regulated PTM networks. Rather than postulating a novel transporter, this model introduces a regulatory layer linking sulfur and methyl-group flux to membrane energetics and signaling adaptability. By positioning membrane energetics as an active component of metabolic–redox coordination, this framework advances a systems-level perspective in which methionine dependence emerges from coordinated energetic, metabolic, and signaling processes rather than isolated transporter activity. The hypothesis generates experimentally tractable predictions: perturbation of thiol redox balance, methyl-group flux, ion gradients, or ATP-dependent membrane processes should produce coordinated alterations in methionine uptake dynamics and PTM signaling states. This model provides a foundation for mechanistic investigation and rational therapeutic exploration.

## Introduction

Methionine dependence represents a recurrent and functionally significant metabolic characteristic of malignant cells and has long been associated with proliferative capacity, redox balance, and epigenetic regulation (1, 2). As a central component of one-carbon metabolism, methionine sustains S-adenosylmethionine–dependent methylation reactions and contributes to redox homeostasis through interconnected sulfur metabolic pathways (14, 15). Heightened sensitivity to methionine availability reflects its integrative role in coordinating biosynthetic flux, chromatin remodeling, and survival signaling processes that influence tumor progression and therapeutic response (7, 8, 16, 23).

Experimental studies have demonstrated that altering methionine availability modulates cell cycle progression, molecular signatures, and proliferative behavior across diverse cancer cell types (10–13). These findings indicate that methionine functions not merely as a metabolic substrate but as a regulatory determinant of signaling stability and transcriptional control. Despite these observations, the mechanisms linking methionine influx to broader energetic and redox-regulated signaling systems remain incompletely resolved. Conceptual models have proposed that methionine-dependent regulatory processes may involve ATP-associated membrane-level coordination (9), yet the structural and functional integration of these domains has not been systematically articulated.

Redox-sensitive post-translational modifications—including reversible thiol oxidation and methylation-dependent regulation—serve as dynamic modulators of protein activity and intracellular signaling networks (3, 4, 24). These processes are metabolically coupled, enabling malignant cells to adapt to oxidative stress and nutrient fluctuation. Glutathione- and thioredoxin-dependent buffering systems, frequently upregulated in cancer, further illustrate the biochemical interdependence between sulfur metabolism and redox control (18). Together, these observations underscore the intimate linkage between methionine metabolism and redox-sensitive signaling architecture.

Established amino acid transport systems, particularly LAT1 (SLC7A5) in complex with SLC3A2, account for a substantial proportion of methionine influx in proliferative and malignant contexts, and their expression commonly correlates with aggressive tumor phenotypes (17). Concurrently, metabolic rewiring in cancer extends beyond amino acid transport to encompass membrane expansion, phospholipid turnover, and choline-dependent one-carbon flux (19, 20). However, increased transporter expression and activation of canonical metabolic pathways do not fully explain how methionine flux is dynamically integrated with ATP-dependent membrane energetics and redox-responsive regulatory networks.

Metabolic, redox, and membrane energetic evidence collectively suggests that transporter- and pathway-centric interpretations may be insufficient to explain regulatory coordination occurring at the membrane–metabolism boundary. A unified energetic framework may therefore be required to clarify how methionine availability, ATP-dependent membrane processes, and redox-regulated post-translational signaling are temporally and functionally integrated in malignant cells. This unresolved integration gap provides the conceptual foundation for the hypothesis advanced below.

## Hypothesis

We propose that malignant cells employ a thiol- and methyl-responsive, ATP-associated regulatory interface operating at the membrane–metabolism boundary to coordinate methionine influx with redox-regulated post-translational modification (PTM) networks.

Methionine dependence reflects coordinated regulation among amino acid transport, sulfur and methyl-group metabolism, cellular energetics, and redox-sensitive signaling. Prior experimental and conceptual investigations have indicated that methionine-associated regulatory effects extend beyond substrate availability and may involve ATP-dependent membrane processes (9–13). However, the structural and functional integration of these energetic and metabolic domains has not been systematically defined.

ATP-dependent membrane processes—including maintenance of electrochemical gradients via Na^+^/K^+^-ATPase activity, which also functions as a membrane-associated signaling platform (22), ATP-driven transporters (5, 6), and vesicular trafficking—constitute major energetic nodes in proliferative cells. Concurrently, methionine metabolism sustains sulfur-dependent redox buffering systems and methylation-dependent PTM networks that regulate chromatin architecture, transcriptional programs, and signal transduction. The biochemical interdependence of these systems supports the plausibility of an integrative regulatory interface linking membrane ATP flux with thiol and methyl chemical states.

Within this framework, membrane energetics are conceptualized as dynamic modulators of metabolic–redox coordination rather than passive infrastructure. Perturbations of thiol redox balance, methyl-group availability, ion gradients, or ATP-dependent membrane processes would be predicted to induce coordinated alterations in methionine uptake kinetics and downstream PTM signaling beyond those anticipated by transporter-centric models alone.

**Figure 1:**
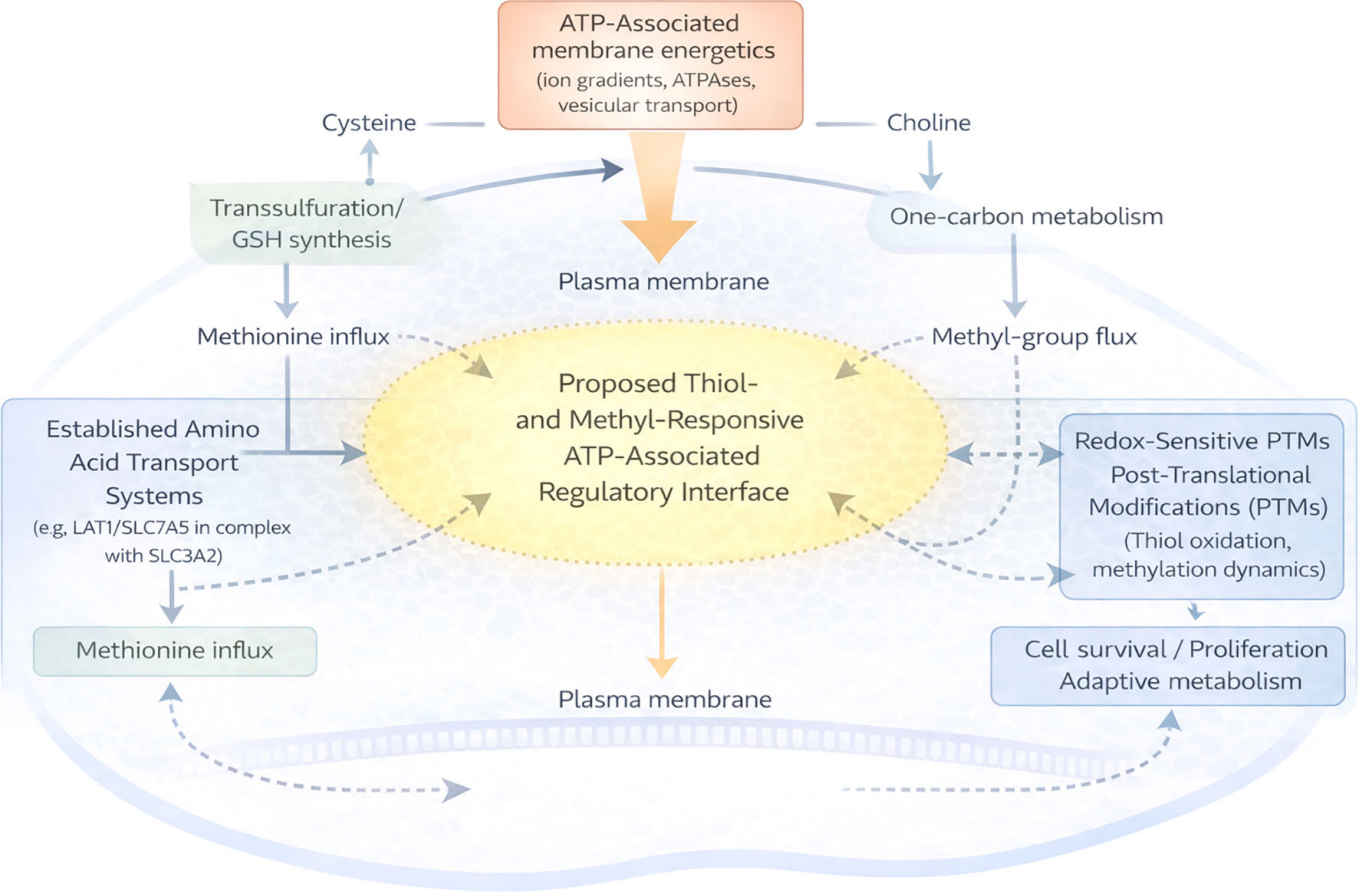
Conceptual schematic illustrating the proposed integration of methionine availability, sulfur and methyl metabolism, ATP-associated membrane energetics, and redox-regulated post-translational modification (PTM) networks in cancer cells. Established amino acid transport systems, including LAT1 (SLC7A5) in complex with SLC3A2, mediate methionine influx. A thiol- and methyl-responsive, ATP-associated regulatory interface is positioned at the membrane–metabolism boundary, where it is proposed to coordinate metabolic inputs with redox-sensitive signaling outputs. Dotted arrows denote hypothesized integrative influences and do not represent established molecular interactions or discrete structural entities.

This hypothesis is experimentally falsifiable. Systematic manipulation of redox buffering capacity, one-carbon flux, and ATP-dependent membrane energetics—individually and combinatorially—should determine whether membrane-level energetic integration modulates methionine-associated signaling stability and metabolic adaptability in malignant cells. A schematic representation of the proposed framework is presented in **Figure 1**.

## Discussion

The framework advanced here integrates methionine uptake, sulfur and methyl metabolism, ATP-associated membrane energetics, and redox-regulated post-translational modification (PTM) networks within a coordinated systems-level architecture. Prevailing models have largely treated these domains as intersecting yet mechanistically separable processes (1–8, 23). The present proposal suggests that malignant cells may couple these processes through an energetic–metabolic interface operating at the membrane–metabolism boundary, thereby enabling dynamic coordination between nutrient flux, redox state, and signaling regulation.

Experimental evidence has demonstrated that methionine availability influences cell-cycle progression, transcriptional reprogramming, and proliferation dynamics in a p53-dependent context (10–13). Furthermore, prior work proposed that methionine analogs may interfere with a membrane-associated disulfide/methyl–ATP–linked regulatory mechanism (9), suggesting functional coupling between sulfur chemistry and ATP-dependent processes. Although these studies did not resolve a discrete structural entity, they indicate that methionine flux, redox balance, and ATP utilization may be functionally integrated at or near the membrane interface. The current framework extends this line of investigation by situating those observations within a broader energetic coordination model.

Biochemically, methionine metabolism occupies a central node linking one-carbon flux, S-adenosylmethionine–dependent methylation, and transsulfuration-derived cysteine production. Through transsulfuration, methionine contributes to glutathione synthesis, sustaining thiol-based redox buffering systems that modulate reversible cysteine oxidation and redox-sensitive signaling (18). In parallel, methyl-group availability regulates chromatin remodeling and transcriptional programs via DNA and histone methylation, thereby influencing cellular identity and proliferative capacity (14, 15). These sulfur- and methyl-dependent processes operate within a context of high ATP demand, membrane biosynthesis, and ion-gradient maintenance characteristic of proliferative cells.

Membrane-associated ATP-dependent systems—including Na^+^/K^+^-ATPase activity, ATP-binding cassette transporters, vesicular trafficking machinery, and phospholipid turnover—constitute major energetic hubs that maintain electrochemical gradients, support membrane expansion, and participate in signal transduction (5, 6, 22). Na^+^/K^+^-ATPase, in particular, has been recognized not only as an ion pump but also as a membrane-associated signaling scaffold influencing kinase cascades and redox-sensitive pathways (22). Within this energetic landscape, fluctuations in thiol redox state or methyl-group flux could theoretically modulate ATP-dependent membrane processes, altering ion homeostasis, vesicular dynamics, or signaling microdomains in a coordinated manner.

Established methionine transport systems such as LAT1 (SLC7A5)–SLC3A2, as well as SLC43 family members, account for methionine influx across diverse malignant contexts (17, 21, 25). However, transporter abundance alone does not necessarily determine methionine-dependent signaling outputs. Transport kinetics, intracellular redox buffering capacity, ATP availability, and membrane potential collectively influence net metabolic flux. The proposed interface therefore does not replace known transporters but posits a regulatory layer through which ATP-dependent membrane energetics and thiol/methyl chemical states may modulate methionine uptake efficiency and downstream PTM stability.

Within this integrative view, methionine dependence may reflect vulnerability in a coordinated energetic–metabolic circuit rather than isolated nutrient addiction. Perturbations in redox buffering systems, methylation flux, ion gradients, or ATP turnover would be predicted to alter methionine-associated signaling in a non-linear and context-dependent manner. Such coupling could contribute to adaptive metabolic plasticity by synchronizing membrane energetics with chromatin regulation and redox-responsive signaling networks.

From a translational perspective, this systems-level framework suggests that combinatorial targeting strategies—simultaneously modulating thiol redox balance, one-carbon metabolism, and ATP-dependent membrane processes—may expose vulnerabilities not evident through transporter inhibition alone. Rather than conceptualizing methionine dependence solely as increased nutrient demand, this model reframes it as a problem of energetic–metabolic integration susceptible to coordinated disruption.

Experimentally, the framework generates specific, testable predictions. Systematic manipulation of Na^+^/K^+^-ATPase activity, ion gradients, vesicular ATP-dependent transport, glutathione/thioredoxin buffering capacity, and methylation flux—individually and in combination—should produce measurable shifts in methionine uptake kinetics, intracellular redox parameters, and PTM-dependent signaling outputs. Integration of metabolic flux analysis, redox proteomics, ion-gradient imaging, and transcriptomic profiling would allow determination of whether membrane-level energetic modulation functionally influences methionine dependence.

If validated, this perspective would reposition methionine vulnerability from a predominantly transporter-centric paradigm to a systems-level phenomenon in which membrane energetics, sulfur metabolism, methyl-group chemistry, and redox-sensitive PTM networks operate as interdependent determinants of signaling stability and adaptive plasticity in malignant cells. Such reframing may refine conceptual models of cancer metabolic regulation and inform future investigations into metabolic–signaling crosstalk in oncologic systems.

## Limitations of the hypothesis

The framework proposed herein reflects the integrative and conceptual scope of this work. It does not identify a discrete transporter, ion channel, or structural complex, nor does it supplant established mechanisms governing methionine transport or one-carbon metabolism. Instead, it advances a functional regulatory interface that may operate in parallel with known systems to coordinate energetic and metabolic inputs.

Direct experimental validation of this integrative mechanism is not provided in the present manuscript. Although the hypothesis is grounded in established biochemical, metabolic, and signaling observations, its validity depends on systematic interrogation through coordinated perturbation of membrane energetics, thiol redox buffering systems, methyl-group flux, and associated signaling outputs. Only through such multi-axis testing can the existence and functional significance of the proposed interface be determined.

The extent and operational characteristics of this integration may vary across tumor types, differentiation states, nutrient availability, and oxidative environments. Such variability would be consistent with the metabolic plasticity and adaptive heterogeneity characteristic of malignant cells. Accordingly, empirical evaluation across diverse biological contexts will be necessary to establish generalizability.

These limitations are intrinsic to the articulation of a testable systems-level hypothesis. The framework is intentionally bounded and remains subject to refinement, constraint, or rejection as experimental evidence accumulates.

## Conclusion

Methionine dependence represents a reproducible and therapeutically relevant metabolic feature of many malignant cell types, yet the mechanisms coordinating methionine uptake with membrane energetics and redox-regulated signaling remain incompletely resolved. The framework advanced here proposes that thiol- and methyl-responsive, ATP-associated membrane processes may constitute an integrative regulatory interface linking methionine availability to redox-dependent post-translational modification networks.

By situating methionine flux within the broader context of membrane energetics, sulfur metabolism, and dynamic signaling regulation, this model reframes methionine dependence from a predominantly transporter-centered phenomenon toward a systems-level problem of energetic–metabolic coordination at the membrane–metabolism boundary. This perspective provides a coherent explanatory structure for aspects of metabolic resilience and adaptive signaling plasticity observed in malignant cells.

If experimentally substantiated, this integrative view may inform future therapeutic strategies aimed not solely at inhibiting nutrient transport, but at modulating coordinated redox buffering, one-carbon metabolism, and ATP-dependent membrane energetics. Systematic investigation of this proposed interface could reveal combinatorial metabolic–signaling vulnerabilities not evident within pathway-isolated models.

Clarifying the mechanistic relationship between membrane energetics and methionine-associated signaling may refine conceptual models of cancer metabolism and expand the foundation for studying energetic–metabolic crosstalk in oncologic systems.
